# Preoperative prediction of language function by diffusion tensor imaging

**DOI:** 10.1007/s40708-017-0064-8

**Published:** 2017-05-04

**Authors:** C. F. Freyschlag, J. Kerschbaumer, D. Pinggera, T. Bodner, A. E. Grams, C. Thomé

**Affiliations:** 1 Department of Neurosurgery, Medical University of Innsbruck, Anichstrasse 35, 6020 Innsbruck, Austria; 2 Department of Neurology, Medical University of Innsbruck, Anichstrasse 35, 6020 Innsbruck, Austria; 3 Department of Neuroradiology, Medical University of Innsbruck, Anichstrasse 35, 6020 Innsbruck, Austria

**Keywords:** Diffusion-tensor imaging, Prediction of function, Awake surgery, Eloquent glioma surgery, Functional MRI

## Abstract

For surgery of eloquent tumors in language areas, the accepted gold standard is functional mapping through direct cortical stimulation (DCS) in awake patients. Ever since, neuroscientists are searching for reliable noninvasive detection of function in the human brain, with variable success. The potential of diffusion tensor imaging (DTI) in combination with computational cortical parcellation to predict functional areas in language eloquent tumors has not been assessed so far. We present a proof-of-concept report involving awake surgery for a temporodorsal tumor. Postoperatively, the imaging was extensively studied and a predictive value of multimodal MR imaging for the possible extent of resection was analyzed. After resection using DCS, the extent of resection and functional outcome were correlated with the processed imaging. Preoperative imaging of our patient was taken to compute the lesion volume as a seed for tractography (DTI) and combined with a tractography of the entire hemisphere. For better spatial resolution, an elastic image fusion was performed to correct the distortion of DTI data. After subtotal resection and imaging analysis, the status of the superior part of the lesion could be identified and predicted as functional cortex. There was a strong correlation between the tumor remnant during surgery and the imaging parameters of DTI connectivity of the eloquent tissue. A combination of complex DTI processing may be able to predict function in a patient suffering eloquent brain tumors and thus allow estimation of extent of resection.

## Introduction

Functional resection of brain tumors is key to achieve both, maximized extent of resection and a maintained quality of life for the patients. The gold standard, to maintain neurological function while resection, is the direct stimulation of cortical brain [1–9]. The goal of direct stimulation in awake brain mapping is to optimize the benefit/risk ratio of surgery by avoiding any damage to crucial structures while increasing the extent of resection. To achieve these aims, it is useful to take advantage of modern neuropsychological testing and neuroradiological imaging in combination with direct cerebral mapping, in order to study the individual anatomical and functional organization of the brain. The trend toward less invasive surgery emphasized preoperative planning including different techniques for noninvasive mapping. Functional MRI (fMRI) is available in most centers and could address several brain tasks through the selection of paradigms tested. The sensitivity of fMRI for language investigation is at lowest 59% [10].

Navigated transcranial magnetic stimulation (nTMS) has shown to obtain a high sensitivity and specificity for prediction of functional areas of the brain [11, 12]. Although this technique has demonstrated safety and efficacy—large investments are needed to obtain the technical infrastructure for nTMS. Therefore, we aimed to analyze the potential of diffusion tensor imaging (DTI) and consecutive cortical parcellation to identify localization of language.

Diffusion tensor imaging has shown its reliability in white matter tract visualization, although it represents a rigid mathematical model. In this proof-of-concept study, we analyzed imaging with respect to the functional connectivity of the cortical area involved by a low-grade glioma. The patient underwent awake craniotomy with speech mapping. After resection, the preoperative imaging was analyzed and correlated with the resectable amount of tumor.

## Methods

Our routine MRI low-grade glioma protocol includes T1 w/o contrast, T2, FLAIR, SWI, DWI, MR perfusion, MR spectroscopy and 31phosphorus MR spectroscopy. Within DWI, we routinely perform DTI sequences containing 64 directions.

Imaging was done with a 3.0 T MR scanner (Siemens TRIO, Siemens, Germany). The B-value was set for 1000. Finally, 38 slices with a thickness of 3 mm and 0.75 × 0.75 mm pixel width were used. Preprocessing was done as follows: eddy current and motion correction (b-matrix rotation) with reference B0 averaged from multiple B0 and noise-level estimation (PCA-based) and denoising. To enhance spatial resolution, an elastic image fusion algorithm was used to correct the present distortion of DWI. Final image material underwent averaging over repetitions. After preprocessing, the tumor volume was segmentated manually on T2 and FLAIR images. We used standard software for image processing and neuronavigation (iPlan, version 3.2, BrainLAB Inc., Feldkirchen, Germany) including two prototype sections (elastic fusion, fiber tracking) which are currently under licensing procedure.

After segmentation and preprocessing, fiber tracking was done deterministically from the later surgical access zone with a minimum fractional anisotropy (FA) around 0.1 and a minimum fiber length of 10 mm. The resulting connections were then used as source for automatical parcellation of the tumor volume with respect to regions connecting with white or gray matter structures of interest at different points.

**Fig. 1:**
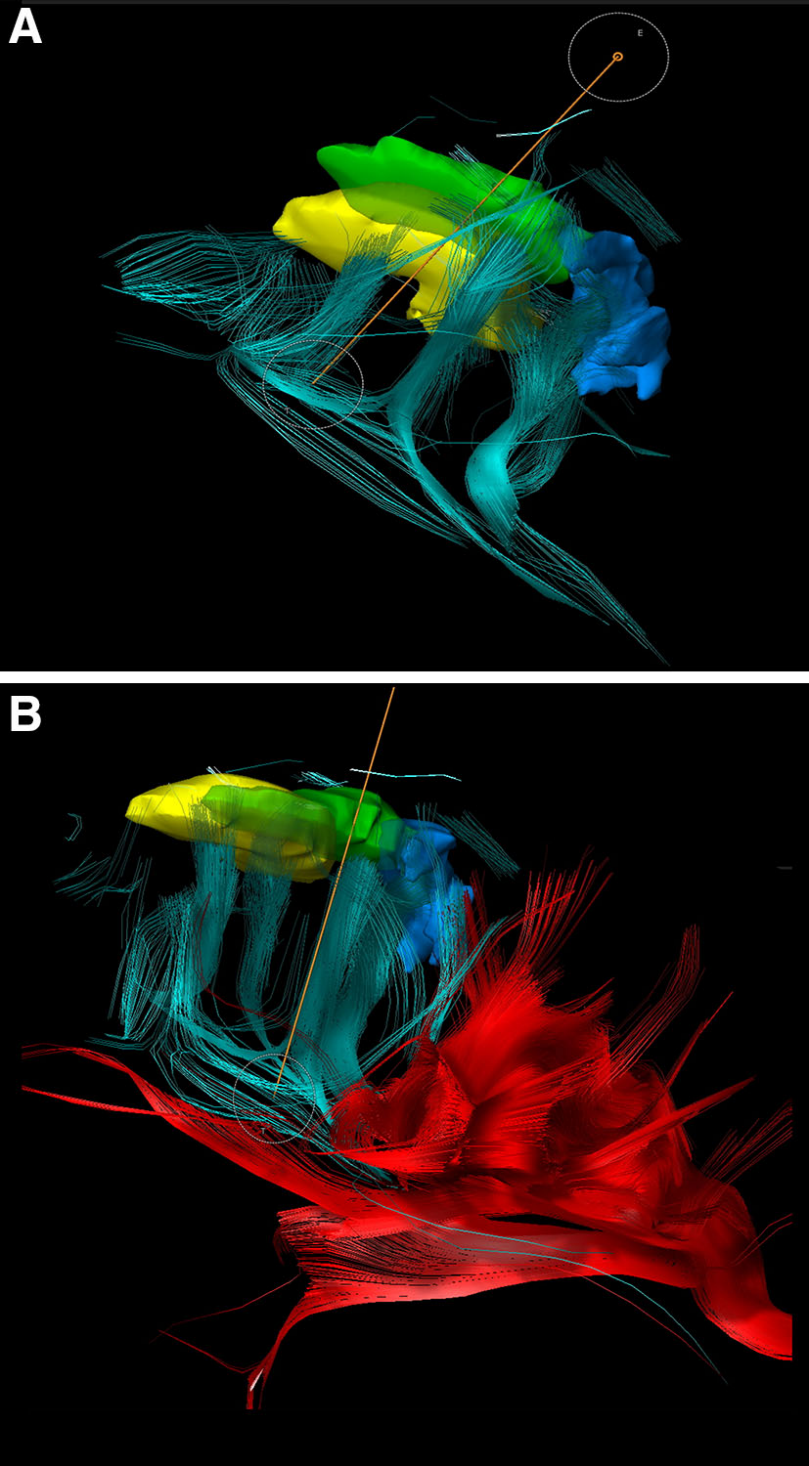
3D reconstruction of fiber tracking showing the parcellated cortical areas with corresponding connection fibers. **a** Fibers between parcellated cortical areas. **b** Additional tracking of the arcuate fascicle (as a part of SLF) showing the direct connectivity to the cortex segmentated in *green*. (Color figure online)

 Postoperative imaging was compared with the above-mentioned processed imaging to determine the extent of resection and a post hoc resection probability analysis (Fig. 1).

## Results

**Fig. 2:**
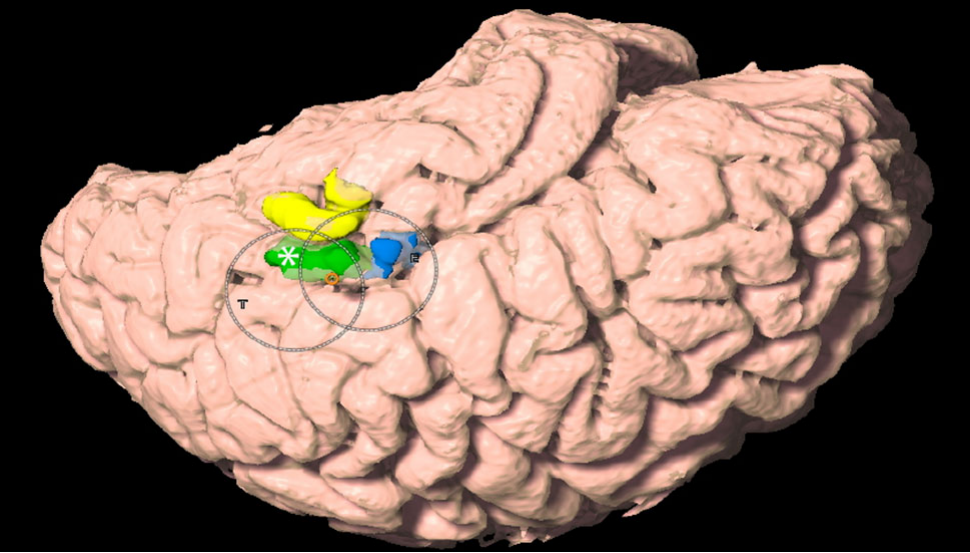
Whole brain surface rendering showing the parcellated pathological areas divided by their connectivity to the fiber tracts. *Green* represents the area of direct connection of the lesion to the SLF via arcuate fascicle. (Color figure online)

**Fig. 3:**
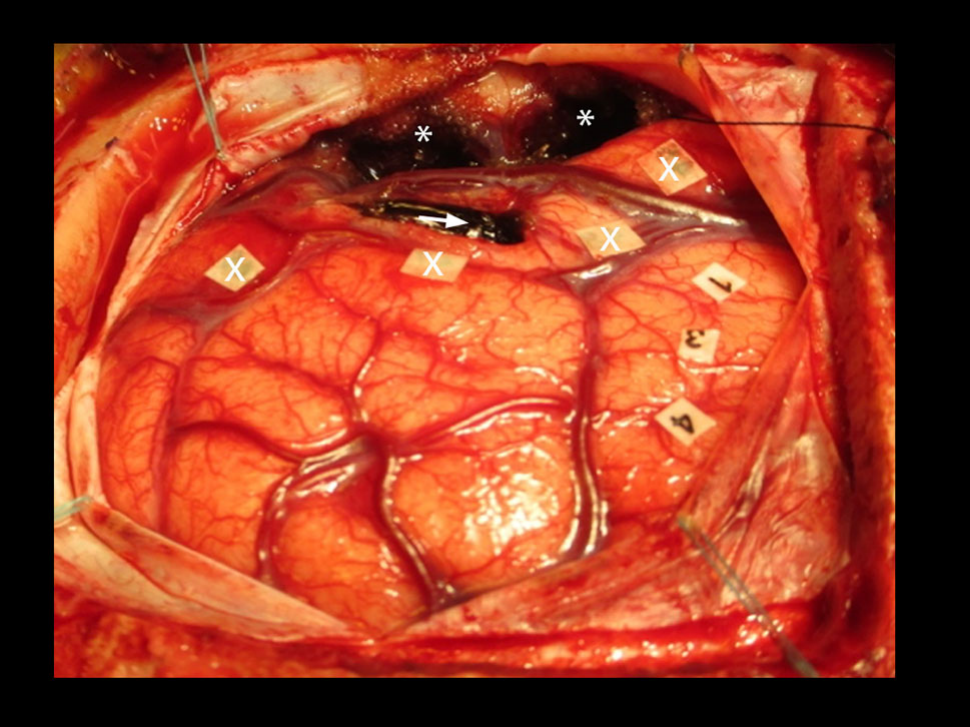
Intraoperative photograph of the resected tumor (*), sonographic tumor boarders (*X*) and speech eloquence during direct cortical stimulation (numbers 1, 3 and 4). The *arrow marks* the area not amenable for resection

The imaging protocol and processing was applied postoperatively to the imaging of a right-handed female patient with a tumor located in the dorsal left temporal lobe. She showed definite language dominance for the left hemisphere and underwent awake surgery with DCS. Subtotal resection could be achieved due to the eloquence of the most superior part of the tumor, shown in our 3D rendered image (see Fig. 2). While starting a resection attempt of the functional area, the patient developed immediate semantic paraphasia leading to discontinuation of the resection. Intraoperative photography (see Fig. 3) depicts the volume of tumor resected.

Postoperative imaging analysis leads to a similarity index of fiber properties, which was used to determine parcellation boundaries within the total volume of the lesion. The resulting fibers connected to relevant systems in different ways. The connection of known semantic and phonetic pathways was analyzed, showing that the superior longitudinal fascicle (SLF) connectivity inserted directly in the green area of the tumor (see Fig. 1) via the arcuate fascicle.

## Discussion

The intraoperative direct cortical simulation of speech eloquent brain tissue represents the standard of care in surgery of eloquent lesions [2, 13, 14]. The value of extensive preoperative functional imaging remains debatable, although it is done by many centers.

It is crucial to underline that functional neuroimaging methods suffer various limitations, and are not yet reliable at the individual scale, mainly because they represent biomathematical reconstructions, whose results may change according to the used model [15].

Recently, DTI was also found to be reliable and predictive for functional recovery in patients with ischemic stroke [16], but up to now there has been no report of functional correlation of DTI connectivity with resectability of cortical tissue in low-grade glioma surgery. Interestingly, functional connectivity has been addressed in several studies by the Montpellier group [17], but functional prediction through direct correlation with preoperative DTI connectivity was not yet described. It is obvious that the presence of fiber tracts within the brain white matter does not necessarily represent the functional connectivity of brain areas. According to De Benedictis and Duffau function [2], the hodotopical organization of elaborate does take influence from several streams of information, leading to synchronized cortical activity. Our analysis does not counteract their hypothesis, and it depicts that language function is strongly bound to cortical areas in relation to fiber connectivity.

Fiber tracking is used for access planning and development of resection strategies in supratentorial, perirolandic or thalamic tumors. The mathematical algorithm underlying the technique of tensor tractography is usually a non-functional, rigid algorithm. Given the fact that DTI is subject to massive distortions [18], the integration of distorted tractography to the intraoperative neuronavigation inevitably leads to imprecision.

Overall, these findings pose the question of the necessity that preoperative tumor assessment should be based on connectivity to other gray matter structures (e.g., subcortical and cortical). Future parcellation can easily be based on connectivity to already known tracts (e.g., SLF). It is a matter of ongoing research to combine pre-segmentation and functional results with more functional imaging (e.g., resting-state fMRI).

In our technical proof-of-concept report, we could demonstrate the feasibility of DTI in combination with cortical parcellation. Our study is limited by its retrospective evaluation and the deterministic approach based on the lesion. Prospective trials are ongoing and will further determine the value of tractography in combination with direct cortical stimulation in surgery for eloquent brain tumors. With the present modality, the prediction of function via DTI seems promising.

## Conclusion

Preoperative tractography, based on the parcellation of cortical structures, is nicely reflecting the underlying white matter connectivity. Furthermore, it was possible to validate and even enhance direct intraoperative stimulation under awake conditions.
